# Perceptions of Patient-Clinician Communication Among Adults With and Without Serious Illness

**DOI:** 10.1001/jamanetworkopen.2025.0365

**Published:** 2025-03-10

**Authors:** Carine Davila, Sarah Nouri, Stephanie H. Chan, Brian Feltz, Anna Gosline, Zamawa Arenas, Jane Kavanagh, Joanna Paladino, Lindsay A. Dow, Vicki A. Jackson, Rebecca Sudore, Christine S. Ritchie, Elizabeth Lindenberger

**Affiliations:** 1 Division of Palliative Care and Geriatric Medicine, Department of Medicine, Massachusetts General Hospital and Harvard Medical School, Boston; 2 Center for Aging and Serious Illness, Mongan Institute, Massachusetts General Hospital, Boston; 3 Division of Palliative Medicine, Department of Medicine, University of California, San Francisco; 4 Massachusetts Coalition for Serious Illness Care, Boston; 5 Blue Cross Blue Shield of Massachusetts, Boston; 6 3D Research Partners LLC, Harvard, Massachusetts; 7 Flowetik, Boston, Massachusetts; 8 Division of Geriatrics, Department of Medicine, University of California, San Francisco

## Abstract

**Importance:**

High-quality, person-centered patient-clinician communication is critical in health care and may be less effective for patients with serious illness. Little is understood about differences in patient-clinician communication experiences of adults with and without serious illness.

**Objectives:**

To determine whether perceptions of patient-clinician communication experiences differ between adults with and without serious illness.

**Design, Setting, and Participants:**

This population-based cross-sectional survey was fielded from April 20 to May 31, 2021, and data were analyzed from January 27, 2023, to December 10, 2024. Participants included a nationally representative sample of US English- or Spanish-speaking adults, including people from historically marginalized groups (eg, Black and Hispanic or Latino individuals, people with low income), responding to an online or telephone survey.

**Exposure:**

Participants were categorized by serious illness status. Participants with serious illness replied yes to (1) having a diagnosis from a list of medical conditions and (2) reporting feeling sicker or having decreased functionality during the last year.

**Main Outcomes and Measures:**

The survey asked about community partner–derived measures of patient-clinician communication experiences, including trusting clinicians, feeling afraid to speak up, and being unsure about next steps. Multivariable logistic regression models were used to estimate the association of serious illness with these communication experiences, adjusting for sociodemographic characteristics. Percentages were weighted according to the National Opinion Research Center’s statistical weighting methods to account for differences in nonresponse.

**Results:**

Of 6126 individuals invited, 1847 (30.2%) completed the survey and were included in analysis (mean [SD] age, 48.4 [17.5] years); 950 (51.8%) identified as female; 191 (11.9%) identified as Black and 287 (16.7%) as Hispanic; and 434 (17.8%) had an annual income less than $30 000 (here called low income). Among respondents, 363 participants (18.5%) had serious illness (mean [SD] age, 50.2 [18.1] years; 218 [64.5%] female; 34 [12.4%] Black; 54 [16.4%] Hispanic; 131 [27.3%] with low income), and 1484 (81.5%) had no serious illness (mean [SD] age, 48.0 [17.4] years; 732 [48.9%] female; 157 [11.8%] Black; 233 [16.7%] Hispanic; 303 [15.6%] with low income). Compared with adults without serious illness, adults with serious illness were more likely to report leaving a visit unsure about next steps (adjusted odds ratio [AOR], 2.30; 95% CI, 1.62-3.27); being afraid to ask questions or speak up (AOR, 2.18; 95% CI, 1.55-3.08); believing they were talked down to or made to feel inferior (AOR, 1.90; 95% CI, 1.24-2.91); and believing that they were treated unfairly by clinicians (AOR, 3.26; 95% CI, 2.43-4.38).

**Conclusions and Relevance:**

In this cross-sectional study, adults with serious illness more often had worse patient-clinician communication experiences. Further research is needed to better understand and develop interventions to improve perceptions of patient-clinician communication experiences for adults with serious illness.

## Key Points

**Question:**

Do patient-clinician communication experiences differ between adults with and without serious illness?

**Findings:**

In this cross-sectional analysis of 1847 survey participants, compared with adults without serious illness, adults with serious illness reported worse communication experiences, and more frequently being treated unfairly, feeling afraid to speak up or ask questions, or leaving appointments unsure about the next steps.

**Meaning:**

These findings suggest that focused effort is needed to improve patient-clinician communication for people facing serious illness.

## Teaser

This cross-sectional study assesses patient-clinician communication experiences for adults with and without serious illness, focusing largely on relational and identity goals.

## Introduction

High-quality communication is critical to the well-being and quality of life of all people, particularly those facing serious illness.^1,2^ Kelley and Bollens-Lund^3^ define serious illness as a health condition that carries a high risk of mortality and either negatively impacts a person’s daily function or quality of life or excessively strains their caregivers. Adults with serious illness can experience distress, isolation, and lost earnings.^4^ They often struggle to get the care, treatment, and support they need in a fragmented health care system.^4^ Adults with serious illness are navigating complicated health care plans, complex decision-making, and frequent health care visits, underscoring why high-quality patient-clinician communication is critical for them.^5,6^ Understanding patient-clinician communication experiences and how they differ in adults with and without serious illness can offer insights to develop and improve clinician-facing and systems-level interventions to improve serious illness care.^7^

High-quality communication is a core competency in clinical education; the Institute of Medicine outlined that health care should be more patient-centered and responsive to patient needs and perspectives, with patient values guiding decision-making.^8,9^ Patient-centered communication was used to describe high-quality communication and included 4 domains: eliciting the patient’s perspective, understanding the psychosocial context, reaching a shared understanding, and sharing power and responsibility.^10^ Evidence demonstrates that patient-centered communication is associated with positive patient outcomes, including improved adherence to treatment plans, increased satisfaction, greater patient participation in decision-making, stronger therapeutic relationships with clinicians, and improvements in psychological distress.^11,12,13,14,15,16,17^ Scott and Van Scoy^18^ propose a framework that we use in this report: high-quality clinical communication is achieved when *relational goals* (eg, maintaining mutual trust) and *identity goals* (eg, tailoring communication to the individual patient), both core to patient-centered communication, are balanced with skillful execution of *task goals* (eg, disclosing prognosis). Another focus of communication literature is on shared decision-making, an interpersonal process where the clinician and patient relate to and influence each other to make decisions about the patient’s health care collaboratively.^19,20^ Shared decision-making, a key component of patient-centered communication, often focuses more on task goals of the clinician disclosing information to the patient, the clinician checking in on the patient’s understanding, and the clinician and patient arriving at a decision together.^21,22,23,24,25^ However, achieving high-quality shared decision-making and communication also requires creating a psychologically safe space for patients to engage in vulnerable discussions and decisions, factors that align with relational and identity goals.^26^ Psychological safety, often studied in the team setting and among health care workers, allows individuals to speak up, share mistakes, and have their voices heard without fear of punishment.^27,28^ These communication frameworks address similar components that comprise high-quality communication: patient-clinician trust (relational goal), medical decision-making (task goal), and respecting individual identity and preferences (identity goal). Much existing communication literature measures how well patient-clinician communication achieves task goals and underfocuses on the importance and measurement of relational and identity goals.

Our study aimed to understand patient-clinician communication experiences for adults with and without serious illness, focusing largely on relational and identity goals. We hypothesized that communication experiences differ between adults with and without serious illness.

## Methods

This cross-sectional study is part of a larger mixed-methods community-informed study by the Massachusetts Coalition for Serious Illness Care previously described.^7^ We conducted a quantitative survey with the National Opinion Research Center (NORC) at the University of Chicago, Chicago, Illinois, using a probability-based panel (AmeriSpeak).^29,30^ The NORC Institutional Review Board (IRB) oversaw survey recruitment, administration, and written informed consent obtained from all participants. The Harvard Longwood Campus IRB approved the survey instrument and analysis. This study followed the relevant portions of the Strengthening the Reporting of Observational Studies in Epidemiology (STROBE) reporting guideline.

### Setting and Participants

In brief, NORC’s probability-based panel uses multistage sampling to construct a panel of 35 000 households covering a sample frame of 97% of the residential US. Households are recruited through a rigorous process using mail, telephone, and in-person approaches to ensure that hard-to-reach populations are represented.^7,31^ Panelists were invited to participate in the survey from April 20 to May 31, 2021. The survey was available in English or Spanish, online or by telephone. Enrollment targets were set at a minimum of 100 respondents for specific groups: people with annual income less than US $50 000 per year, people who identify as Black, people who identify as Hispanic or Latino (hereafter called Hispanic), adults 65 years or older, people with disability (self-identified and/or answering yes to any of 6 questions about function from the American Community Survey),^32,33^ and people with serious illness (see Main Measures section). People younger than 18 years were excluded. We also excluded people who received a diagnosis of Alzheimer disease, dementia, or memory loss, as they may not have understood the questions and answer choices.

### Main Measures

The primary exposure variable was whether the participant was identified as having a serious illness (yes or no). We identified adults with serious illness by their reporting yes to 2 questions. First, they had a diagnosis of any of the following: diabetes; asthma, lung disease, emphysema, or chronic obstructive pulmonary disease; heart disease or stroke; cancer; depression, anxiety, or other serious mental health problems; or chronic kidney disease or kidney failure. Second, over the last year, they reported feeling sicker and that it is harder to do their normal levels of work and activity.

Several of us previously engaged community partners to better understand what matters to people around serious illness care and communication.^7^ Community partners included patients and clinicians at a federally qualified health center and leading serious illness care organizations nationally. The investigators asked about concerns around health care and communication, how to ask about people’s health care communication experiences, and what needed to change to improve people’s health care experiences. These invaluable insights informed survey instrument development, including the patient-centered communication measures described herein, largely reflecting relational and identity goals.

The primary dependent variables included patient-reported measures of communication framed by the 3 goals of high-quality clinical communication: relational, task, and identity goals. We focused on relational goals (eg, trust in your clinician to do what is right for you) and identity goals (eg, being treated with dignity and respect, believing you are talked down to), and also included task goals (eg, left a visit unsure about discussion or next steps). We dichotomized the 4-point Likert scale responses to these questions to reflect the relatively frequent or infrequent occurrence of these experiences (see eTable 1 in Supplement 1 for details).

Baseline participant demographic information (age, gender, race and ethnicity, income, metropolitan area, geographic region, and marital status) was collected by NORC. Participants’ race and ethnicity were self-reported upon enrollment to the probability-based panel^30^ and included Hispanic, non-Hispanic Asian, non-Hispanic Black, non-Hispanic White, and non-Hispanic other (including multiple race or other). These demographic data were the covariables used in subsequent analyses.

### Data Collection Tools

Based on the social-ecological model, the survey had questions across intrapersonal, interpersonal, and systems-level domains.^34^ The primary variables of interest for this study draw from the interpersonal domain, specifically on patient-clinician communication experiences mostly related to relational and identity goals (the full survey can be found elsewhere).^7^

### Statistical Analysis

We conducted analyses from January 27 to July 24, 2023, with additional analyses until December 10, 2024. NORC applied statistical weighting to account for differences between the survey sample and US Census benchmarks and for interactions of age with gender, age with race and ethnicity, and race and ethnicity with gender.^31^ The weighted data reflecting the US population of adults 18 years and older were used in analyses. We used the χ^2^ test for categorical variables and unpaired *t* test and analysis of variance for continuous variables to compare sociodemographic covariables and patient-clinician communication measures. We performed multivariable logistic regressions adjusting for sociodemographic covariables to assess associations between serious illness status and patient-clinician communication outcomes. Given the exploratory nature of this effort, we maintained a significance threshold of *P* < .05 (2-sided). Analyses were conducted using SPSS, version 29 (IBM Corp). Percentages were weighted according to NORC’s statistical weighting methods to account for differences in nonresponse.

## Results

### Participant Characteristics

Of 6126 panelists invited, 1854 (30.3%) completed the survey. People who reported a diagnosis of Alzheimer disease, dementia, or memory problems (n = 7) were excluded, resulting in 1847 (30.2%) included in analyses. As shown in the Table, participants had a mean (SD) age of 48.4 (17.5) years. A total of 950 participants (51.8%) identified as female, 865 (46.2%) identified as male, and 22 (1.0%) identified as transgender or nonbinary. Race and ethnicity included 287 participants (16.7%) who identified as Hispanic, 50 (6.5%) as non-Hispanic Asian, 191 (11.9%) as non-Hispanic Black, 1215 (61.3%) as non-Hispanic White, and 104 (3.7%) as other race or ethnicity. A total of 1091 participants (62.0%) were married or living with their partner, and 1562 (85.4%) lived in a metropolitan area. There was socioeconomic diversity in participants’ annual household income: 434 (17.8%) reported less than $30 000; 524 (25.1%) reported $30 000 to less than $60 000; 463 (30.2%) reported $60 000 to less than $100 000; and 426 (26.9%) reported $100 000 or greater. The sample reflected geographic diversity, with the most reported region of residence being the South (627 [38.1%]).

**Table.** Participant Characteristics, Categorized by Serious Illness Status

Characteristic	No. of participants (weighted %)^a^			*P* value
	Overall (n = 1847 [100%])	Serious illness (n = 363 [18.5%])	No serious illness (n = 1484 [81.5%])	
Age, mean (SD), y	48.4 (17.5)	50.2 (18.1)	48.0 (17.4)	.03
Age category, y				
18-24	70 (7.0)	12 (4.6)	58 (7.5)	.11
25-34	415 (22.6)	70 (22.4)	345 (22.6)	
35-44	292 (17.3)	57 (16.4)	235 (17.5)	
45-54	251 (12.7)	44 (12.3)	207 (12.8)	
55-64	354 (18.7)	68 (17.3)	286 (19.1)	
65-74	323 (14.9)	72 (17.7)	251 (14.2)	
≥75	142 (6.8)	40 (9.2)	102 (6.2)	
Gender^b^				
Female	950 (51.8)	218 (64.5)	732 (48.9)	<.001
Male	865 (46.2)	134 (33.7)	731 (49.1)	
Transgender or nonbinary	22 (1.0)	9 (1.6)	13 (0.9)	
Race and ethnicity				
Hispanic	287 (16.7)	54 (16.4)	233 (16.7)	.11
Non-Hispanic Asian	50 (6.5)	9 (3.9)	41 (7.1)	
Non-Hispanic Black	191 (11.9)	34 (12.4)	157 (11.8)	
Non-Hispanic White	1215 (61.3)	237 (62.1)	978 (61.1)	
Non-Hispanic other^c^	104 (3.7)	29 (5.2)	75 (3.3)	
Household income, US $				
<30 000	434 (17.8)	131 (27.3)	303 (15.6)	<.001
30 000 to <60 000	524 (25.1)	113 (31.5)	411 (23.7)	
60 000 to <100 000	463 (30.2)	74 (27.9)	389 (30.7)	
≥100 000	426 (26.9)	45 (13.4)	381 (30.0)	
Metropolitan area	1562 (85.4)	293 (81.4)	1269 (86.3)	.02
Geographic region				
Northeast	255 (17.3)	36 (11.8)	219 (18.6)	.02
Midwest	488 (20.7)	100 (20.9)	388 (20.6)	
South	627 (38.1)	131 (42.4)	496 (37.1)	
West	477 (24.0)	96 (24.8)	381 (23.8)	
Marital status				
Married or living with partner	1091 (62.0)	203 (62.1)	888 (61.9)	.99
Previously married (divorced, separated, or widowed)	365 (16.5)	86 (16.7)	279 (16.5)	
Never married	391 (21.5)	74 (21.3)	317 (21.6)	
Importance of faith or spirituality^b^				
Very important	770 (41.2)	147 (44.1)	623 (40.6)	.18
Somewhat important	444 (25.7)	87 (22.8)	357 (26.4)	
Not too important	248 (14.1)	56 (15.2)	192 (13.8)	
Not important at all	339 (16.7)	68 (17.0)	271 (16.7)	
Confidence managing health problems^b^				
I don’t have any health problems	132 (7.3)	4 (0.7)	128 (8.7)	<.001
Not very confident	182 (11.3)	78 (20.8)	104 (9.2)	
Somewhat confident	977 (52.6)	215 (60.0)	762 (50.9)	
Very confident	555 (28.8)	66 (18.5)	489 (31.1)	

^a^ Percentages are weighed according to the National Opinion Research Center’s statistical weighting methods to account for differences in nonresponse.

^b^ Data were missing for fewer than 1% for gender identity, fewer than 3% for importance of faith or spirituality, and fewer than 1% for confidence managing health problems.

^c^ Includes respondents who selected multiple race or other.

Based on our definition of serious illness, 363 participants (18.5%) were identified as having a serious illness (Table). Compared with adults without serious illness (1484 [81.5%]), adults with serious illness were slightly older (mean [SD] age, 50.2 [18.1] vs 48.0 [17.4] years; *P* = .03), were more likely to identify as female (218 [64.5%] vs 732 [48.9%]; *P* < .001), and were more likely to have a lower household income (eg, <$30 000; 131 [27.3%] vs 303 [15.6%]; *P* < .001). Participants with serious illness were similarly likely as those without serious illness to identify as non-Hispanic Black (34 [12.4%] and 157 [11.8%], respectively) or Hispanic (54 [16.4%] and 233 [16.7%], respectively; *P* = .11). People with serious illness were less likely to live in a metropolitan area (293 [81.4%] vs 1269 [86.3%]; *P* = .02), less likely to be from the Northeast (36 [11.8%] vs 219 [18.6%]; *P* = .02), and less likely to be very confident managing their own health problems (66 [18.5%] vs 489 [31.1%]; *P* < .001).

### Patient-Centered Communication Measures

The Figure summarizes the proportions and the adjusted odds ratios (AORs) of patient-centered communication measures by relational, task, and identity goals across respondents by serious illness status. The unadjusted ORs and AORs were similar across the communication measures (eTable 2 in Supplement 1). In terms of relational goals, adults with serious illness were less likely than those without serious illness to trust clinicians to do what is right for them (244 [68.7%] vs 1108 [77.4%]; AOR, 0.70; 95% CI, 0.53-0.92; *P* = .01). Compared with adults without serious illness, adults with serious illness were nearly twice as likely to be afraid to speak up or ask questions (73 [21.4%] vs 144 [11.2%]; AOR, 2.18; 95% CI, 1.55-3.08; *P* < .001) and nearly 3 times as likely to report having been treated unfairly by clinicians in the past year (108 [30.6%] vs 179 [11.8%]; AOR, 3.26; 95% CI, 2.43-4.38; *P* < .001).

**Figure.**
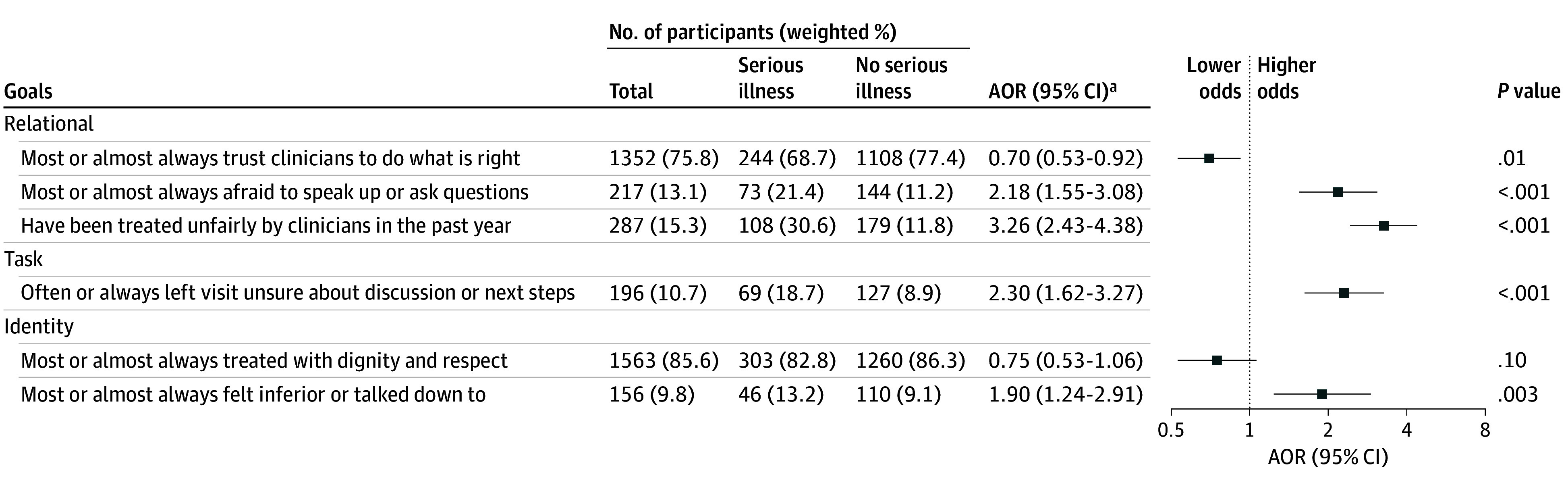
Multivariable Analysis of Odds of Perceptions of Patient-Clinician Communication Experiences Across Adults With and Without Serious Illness Relational goals include building rapport and establishing mutual trust; task goal, disclosing medical information such as prognosis; and identity goals, tailoring conversations to the individual needs of the patient and family. Patients without serious illness served as the reference group. Adjusted odds ratios (AORs) of perceptions of patient-clinician communication experiences by serious illness status, with 95% CI. Effect estimates were derived using logistic regression adjusting for age, gender, race and ethnicity, income, metropolitan area, region, and marital status. ^a^Participants with missing outcome data (<3%) were excluded from multivariable analysis.

We queried a single task goal: adults with serious illness were more than twice as likely as those without serious illness to leave a health care visit unsure about the discussion or next steps (69 [18.7%] vs 127 [8.9%]; AOR, 2.30; 95% CI, 1.62-3.27; *P* < .001).

In terms of identity goals, adults with serious illness were more likely to report clinicians talking down to them or making them feel inferior (46 [13.2%] vs 110 [9.1%]; AOR, 1.90; 95% CI, 1.24-2.91; *P* = .003). There was no statistical difference between adults with and without serious illness reporting that clinicians treated them with dignity and respect (303 [82.8%] vs 1260 [86.3%]; AOR, 0.75; 95% CI, 0.53-1.06; *P* = .10).

## Discussion

In this cross-sectional study, we hypothesized that communication experiences differed between adults with and without serious illness. To our knowledge, this study is the first to examine the communication experiences of adults with and without serious illness in a nationally representative sample across age groups, including younger (<65 years) and Medicare-eligible older (≥65 years) adults. In this survey, we observed that having serious illness was associated with worse communication experiences such as believing they were talked down to, being afraid to speak up or ask questions, and believing they were treated unfairly.

Adults with serious illness reported worse communication experiences in the relational goal dimension. Compared with those without serious illness, adults with serious illness were less likely to trust clinicians to do what is right for them, more likely to be afraid to speak up or ask questions, and more likely to report being treated unfairly by clinicians in the past year, which all can erode trust in clinicians. These findings are important given the evidence outlining how trust and patient-clinician therapeutic relationships lead to better patient outcomes.^16,28,35,36,37^ In this study, more than 15% of respondents reported feeling that they had been treated unfairly by clinicians in the past year; adults with serious illness were nearly 3 times as likely as those without serious illness to report unfair treatment, even after controlling for sociodemographic factors. Adults with serious illness have frequent health care visits during which they hear and must process medical information that can be emotionally charged.^1,38^ They are often asked to make complex medical decisions in the context of adjusting to difficult and declining health.^1^ Even under the best of circumstances, these conversations may at times feel emotionally distressing and challenging for both patients and clinicians. The emotional weight of these conversations may also influence patients’ perceptions of communication experiences. Skilled communication that includes empathic listening, exploration, and responding to patient emotions can help foster adaptive coping to illness and improve the patient experience.^38,39,40,41^ However, care and communication are more challenging when concerns about trust in clinicians, fears around asking questions, and worries about unfair treatment arise.

Similarly, adults with serious illness reported worse communication experiences than those without serious illness around identity goals. Adults with serious illness were twice as likely to believe that clinicians talked down to them or made them feel inferior. Adults with serious illness were less likely to believe clinicians treated them with dignity and respect, though this difference was not significant. These experiences may be connected to the power dynamics between patient and clinician, which studies show influence patient outcomes.^42,43^ When a patient feels talked down to, it reinforces a traditional power dynamic where the clinician holds medical information that the patient depends on.^44^ Cox and Fritz^45^ argue that language that belittles or places blame on patients affects how patients perceive their health and clinicians’ attitudes toward patients and the care and treatments offered. We posit that the belief that one is being talked down to or made to feel inferior should never occur, yet nearly 10% of all respondents cited this perception in our study.

Adults with serious illness also reported worse communication experiences related to the task goal we queried. They were more than twice as likely to report leaving a health care visit unsure about the discussion or next steps. To be sure, adults with serious illness have more complex care plans and more to remember. This finding nevertheless suggests that, coupled with worse experiences in relational and identity-related communication goals, adults with serious illness also have worse outcomes around task goals, such as communicating information about the plan and next steps.

Adults with serious illness reported worse communication experiences across relational, task, and identity goals, so we surmise they are less likely to achieve high-quality communication found with successfully juggling all 3 goals. This finding represents a key challenge, as adults with serious illness not only grapple with complex medical information and care plans but also with their own emotions around how their illness may impact their daily lives and identities. Acknowledging patients’ emotions and affect (aligned to relational and identity goals) is essential in delivering serious news alongside compassionate prognostic disclosure (task goal).^38^ Clinicians must attend to both emotions and medical information sharing to achieve high-quality communication.^38^ People with serious illness also seemingly do not perceive psychological safety in clinical interactions, given the high levels of fear associated with speaking up or asking questions. Though psychological safety has been studied in clinical team communication,^46,47,48^ this finding underscores the need to better understand psychological safety in patient-clinician communication. Our study highlights the value of examining patient experience measures around relational and identity goals in communication, such as safety to ask questions, the impression of being talked down to, or believing they are treated unfairly. Future studies are needed to explore how these communication experiences impact patient health outcomes and explore potential clinician and health systems interventions that could improve these experiences and outcomes.

Our findings are consistent with other studies that show that people with serious illness and multiple chronic medical conditions have increased emotional and psychological distress and experience poor coordination across the health care system.^4,49^ Our finding that serious illness status is associated with worse patient-clinician communication experiences is similar to that seen for people with a disability identity, separately analyzed from the same survey data.^50^ It is also possible that a person with multiple marginalized identities may be particularly vulnerable to these worse communication experiences, reflecting the impact of intersectionality. Cumulative communication experiences influence trust in their clinicians and future communication and engagement in the health care system. It is important that clinicians and health care systems understand these experiences to inform how they can improve high-quality person-centered care and communication to all patients, including those with and without serious illness.

### Limitations

This study has several limitations. First, the survey’s response rate was 30.2%, potentially subjecting these findings to nonresponse bias. However, given the intentional recruitment approach for NORC’s probability-based panel that results in a US Census–representative panel, the demographic composition of survey responders and nonresponders was similar.^31^ Second, the survey does not capture the perspectives of people with limited English proficiency who speak a language other than Spanish. Third, we asked participants to report their experience with clinicians in general rather than about specific clinical experiences or patient-clinician pairings. They may be more likely to recall negative experiences than positive experiences. By contrast, the fact that it is not tied to a specific clinician may enable participants to respond more honestly without the risk of highlighting the shortcomings of their own clinicians.

## Conclusions

The findings of this cross-sectional study show that adults with serious illness experience inequities in patient-clinician communication compared with those without serious illness, despite being a population for whom patient-centered communication is critical for high-quality care. A person with multiple marginalized identities (based on income, educational attainment, race and ethnicity, spoken language, or disability) likely faces additional pressures around lack of time, worry about getting fair treatment, and patient-clinician power imbalance.^51,52,53^ Serious illness is not a marginalized identity in the typical way; however, our study highlights that having a serious illness is a vulnerable identity that can impact a person’s perceived treatment in the health care system. Adults with serious illness are already ill, can be harder to engage, may feel overwhelmed due to complex clinical plans, and may have emotional responses to their illness journey that impact how they receive information and perceive communication, all of which contribute to a greater patient-clinician power imbalance. This underscores the importance of empathic communication in the care of people with serious illness. Recognizing these inequities is a critical step in improving the quality of care for people with serious illness. These community partner–derived questions offer new opportunities for measuring the quality of patients’ communication experiences. These findings are critical for clinicians and health systems to recognize the need to improve high-quality, person-centered communication for all, particularly those with serious illness. Next steps include examining how these experiences differ by sociodemographic factors, such as race and ethnicity and income levels, and understanding whether these patterns are similar to those seen for adults with serious illness. Further research is needed to understand and develop interventions that promote high-quality, patient-centered communication for all people facing serious illness from diverse patient populations and health care settings.

## Notes

Supplement 1. **eTable 1.** Outcome Measures and Dichotomization **eTable 2.** Perceptions of Health Care Communication Experience Outcomes by Serious Illness Status
eReferences

Supplement 2.
Data Sharing Statement

## References

[1] Bernacki RE, Block SD; American College of Physicians High Value Care Task Force. Communication about serious illness care goals: a review and synthesis of best practices. JAMA Intern Med. 2014;174(12):1994-2003. doi:10.1001/jamainternmed.2014.5271 25330167

[2] Committee on Approaching Death. Addressing Key End of Life Issues; Institute of Medicine. Dying in America: Improving Quality and Honoring Individual Preferences Near the End of Life. National Academies Press; 2015. doi:10.17226/18748 25927121

[3] Kelley AS, Bollens-Lund E. Identifying the population with serious illness: the “denominator” challenge. J Palliat Med. 2018;21(S2)(suppl 2):S7-S16. doi:10.1089/jpm.2017.0548 29125784 PMC5756466

[4] Schneider E, Abrams M, Shah A, Lewis C, Shah T. Health care in America: the experience of people with serious illness. October 2018. Accessed July 21, 2023. https://www.commonwealthfund.org/sites/default/files/2018-10/Schneider_HealthCareinAmerica.pdf

[5] McDonald KM, Sundaram V, Bravata DM, . Closing the Quality Gap: A Critical Analysis of Quality Improvement Strategies. Vol 7: Care Coordination. Agency for Healthcare Research and Quality; 2007. 20734531

[6] Sudore RLMD, Knight SJP, McMahan RD, . A novel website to prepare diverse older adults for decision making and advance care planning: a pilot study. J Pain Symptom Manage. 2014;47(4):674-686. doi:10.1016/j.jpainsymman.2013.05.023 23972574 PMC4111443

[7] Davila C, Chan SH, Gosline A, . Online forums as a tool for broader inclusion of voices on health care communication experiences and serious illness care: mixed methods study. J Med Internet Res. 2023;25(3):e48550. doi:10.2196/48550 38055311 PMC10733833

[8] Van Scoy LJ, Scott AM, Green MJ, . Communication quality analysis: a user-friendly observational measure of patient-clinician communication. Commun Methods Meas. 2022;16(3):215-235. doi:10.1080/19312458.2022.2099819 37063460 PMC10104441

[9] Institute of Medicine Committee on Quality of Health Care in America. Crossing the Quality Chasm: A New Health System for the 21st Century. National Academy Press; 2001.

[10] Epstein RM, Franks P, Fiscella K, . Measuring patient-centered communication in patient-physician consultations: theoretical and practical issues. Soc Sci Med. 2005;61(7):1516-1528. doi:10.1016/j.socscimed.2005.02.001 16005784

[11] Heisler M, Bouknight RR, Hayward RA, Smith DM, Kerr EA. The relative importance of physician communication, participatory decision making, and patient understanding in diabetes self-management. J Gen Intern Med. 2002;17(4):243-252. doi:10.1046/j.1525-1497.2002.10905.x 11972720 PMC1495033

[12] Kaplan SH, Greenfield S, Ware JE Jr. Assessing the effects of physician-patient interactions on the outcomes of chronic disease. Med Care. 1989;27(3)(suppl):S110-S127. doi:10.1097/00005650-198903001-00010 2646486

[13] Stewart M, Brown JB, Donner A, . The impact of patient-centered care on outcomes. J Fam Pract. 2000;49(9):796-804. 11032203

[14] Stewart MA. Effective physician-patient communication and health outcomes: a review. CMAJ. 1995;152(9):1423-1433. 7728691 PMC1337906

[15] Sharkiya SH. Quality communication can improve patient-centred health outcomes among older patients: a rapid review. BMC Health Serv Res. 2023;23(1):886. doi:10.1186/s12913-023-09869-8 37608376 PMC10464255

[16] Pinto RZ, Ferreira ML, Oliveira VC, . Patient-centred communication is associated with positive therapeutic alliance: a systematic review. J Physiother. 2012;58(2):77-87. doi:10.1016/S1836-9553(12)70087-5 22613237

[17] Moseson EM, Wiener RS, Golden SE, . Patient and clinician characteristics associated with adherence: a cohort study of veterans with incidental pulmonary nodules. Ann Am Thorac Soc. 2016;13(5):651-659. doi:10.1513/AnnalsATS.201511-745OC 27144794

[18] Scott AM, Van Scoy LJ. What counts as “good” clinical communication in the coronavirus disease 2019 era and beyond?: ditching checklists for juggling communication goals. Chest. 2020;158(3):879-880. doi:10.1016/j.chest.2020.05.539 32473173 PMC7255318

[19] Sanders JJ, Curtis JR, Tulsky JA. Achieving goal-concordant care: a conceptual model and approach to measuring serious illness communication and its impact. J Palliat Med. 2018;21(S2):S17-S27. doi:10.1089/jpm.2017.0459 29091522 PMC5756461

[20] Légaré F. Shared decision making: moving from theorization to applied research and hopefully to clinical practice. Patient Educ Couns. 2013;91(2):129-130. doi:10.1016/j.pec.2013.03.004 23561249

[21] Barry MJ, Edgman-Levitan S. Shared decision making—pinnacle of patient-centered care. N Engl J Med. 2012;366(9):780-781. doi:10.1056/NEJMp1109283 22375967

[22] Steffensen KD, Vinter M, Crüger D, . Lessons in integrating shared decision-making into cancer care. J Oncol Pract. 2018;14(4):229-235. doi:10.1200/JOP.18.00019 29641952

[23] Shay LA, Lafata JE. Where is the evidence? a systematic review of shared decision making and patient outcomes. Med Decis Making. 2015;35(1):114-131. doi:10.1177/0272989X14551638 25351843 PMC4270851

[24] Agency for Healthcare Research and Quality. The SHARE approach—achieving patient-centered care with shared decisionmaking: a brief for administrators and practice leaders. Updated September 2020. Accessed June 29, 2024. https://www.ahrq.gov/health-literacy/professional-training/shared-decision/tool/resource-9.html

[25] Edwards A, Elwyn G. Shared Decision-Making in Health Care: Achieving Evidence-Based Patient Choice. 2nd ed. Oxford University Press; 2009.

[26] Montori VM, Ruissen MM, Hargraves IG, Brito JP, Kunneman M. Shared decision-making as a method of care. BMJ Evid Based Med. 2023;28(4):213-217. doi:10.1136/bmjebm-2022-112068 36460328 PMC10423463

[27] Fukami T. Patient engagement with psychological safety. Dialogues Health. 2023;3:100153. doi:10.1016/j.dialog.2023.100153 38515810 PMC10953965

[28] Thomas T, Althouse A, Sigler L, . Stronger therapeutic alliance is associated with better quality of life among patients with advanced cancer. Psychooncology. 2021;30(7):1086-1094. doi:10.1002/pon.5648 33547717 PMC9243649

[29] NORC at the University of Chicago. Accessed April 26, 2023. https://www.norc.org/

[30] AmeriSpeak is NORC at the University of Chicago’s panel-based research platform. Accessed April 26, 2023. https://amerispeak.norc.org/

[31] Technical overview of the AmeriSpeak panel: NORC’S probability-based household panel. Updated February 8, 2022. Accessed April 26, 2023. https://amerispeak.norc.org/content/dam/amerispeak/research/pdf/AmeriSpeak%20Technical%20Overview%202019%2002%2018.pdf

[32] Office of the Assistant Secretary for Planning and Evaluation. HHS implementation guidance on data collection standards for race, ethnicity, sex, primary language, and disability status. October 30, 2011. Accessed April 12, 2023. https://aspe.hhs.gov/reports/hhs-implementation-guidance-data-collection-standards-race-ethnicity-sex-primary-language-disability

[33] CDC. Disability Health and Data System. Updated December 18, 2024. Accessed January 29, 2025. https://www.cdc.gov/dhds/datasets/?CDC_AAref_Val=

[34] Crabtree BF, Miller WL. Doing Qualitative Research. Sage Publications; 1992.

[35] Eton DT, Ridgeway JL, Linzer M, . Healthcare provider relational quality is associated with better self-management and less treatment burden in people with multiple chronic conditions. Patient Prefer Adherence. 2017;11:1635-1646. doi:10.2147/PPA.S145942 29033551 PMC5630069

[36] Desta R, Blumrosen C, Laferriere HE, . Interventions incorporating therapeutic alliance to improve medication adherence in Black patients with diabetes, hypertension and kidney disease: a systematic review. Patient Prefer Adherence. 2022;16:3095-3110. doi:10.2147/PPA.S371162 36404799 PMC9673796

[37] Murray B, Tichnell C, Burch AE, Calkins H, James CA. Strength of the genetic counselor: patient relationship is associated with extent of increased empowerment in patients with arrhythmogenic cardiomyopathy. J Genet Couns. 2022;31(2):388-397. doi:10.1002/jgc4.1499 34672408

[38] Jackson VA, Emanuel L. Navigating and communicating about serious illness and end of life. N Engl J Med. 2024;390(1):63-69. doi:10.1056/NEJMcp2304436 38118003

[39] Greer JA, Applebaum AJ, Jacobsen JC, Temel JS, Jackson VA. Understanding and addressing the role of coping in palliative care for patients with advanced cancer. J Clin Oncol. 2020;38(9):915-925. doi:10.1200/JCO.19.00013 32023161 PMC7082158

[40] Jacobsen J, Bernacki R, Paladino J. Shifting to serious illness communication. JAMA. 2022;327(4):321-322. doi:10.1001/jama.2021.23695 34994773

[41] Paladino J, Sanders JJ, Fromme EK, . Improving serious illness communication: a qualitative study of clinical culture. BMC Palliat Care. 2023;22(1):104. doi:10.1186/s12904-023-01229-x 37481530 PMC10362669

[42] Ondenge K, Renju J, Bonnington O, . “I am treated well if I adhere to my HIV medication”: putting patient-provider interactions in context through insights from qualitative research in five sub-Saharan African countries. Sex Transm Infect. 2017;93(suppl 3):e052973. doi:10.1136/sextrans-2016-052973 28736392 PMC5739840

[43] House TR, Wightman A, Rosenberg AR, Sayre G, Abdel-Kader K, Wong SPY. Challenges to shared decision making about treatment of advanced CKD: a qualitative study of patients and clinicians. Am J Kidney Dis. 2022;79(5):657-666.e1. doi:10.1053/j.ajkd.2021.08.021 34673161 PMC9016096

[44] Shutzberg M. The doctor as parent, partner, provider… or comrade? distribution of power in past and present models of the doctor-patient relationship. Health Care Anal. 2021;29(3):231-248. doi:10.1007/s10728-021-00432-2 33905025 PMC8322008

[45] Cox C, Fritz Z. Presenting complaint: use of language that disempowers patients. BMJ. 2022;377:e066720. doi:10.1136/bmj-2021-066720 35477529 PMC9273034

[46] Grailey KE, Murray E, Reader T, Brett SJ. The presence and potential impact of psychological safety in the healthcare setting: an evidence synthesis. BMC Health Serv Res. 2021;21(1):773. doi:10.1186/s12913-021-06740-6 34353319 PMC8344175

[47] Yousaf M, Khan MM, Paracha AT. Effects of inclusive leadership on quality of care: the mediating role of psychological safety climate and perceived workgroup inclusion. Healthcare (Basel). 2022;10(11):2258. doi:10.3390/healthcare10112258 36421582 PMC9690171

[48] Kerrissey MJ, Hayirli TC, Bhanja A, Stark N, Hardy J, Peabody CR. How psychological safety and feeling heard relate to burnout and adaptation amid uncertainty. Health Care Manage Rev. 2022;47(4):308-316. doi:10.1097/HMR.0000000000000338 35135989 PMC9422764

[49] May CR, Cummings A, Myall M, . Experiences of long-term life-limiting conditions among patients and carers: what can we learn from a meta-review of systematic reviews of qualitative studies of chronic heart failure, chronic obstructive pulmonary disease and chronic kidney disease? BMJ Open. 2016;6(10):e011694. doi:10.1136/bmjopen-2016-011694 27707824 PMC5073552

[50] Salinger MR, Feltz B, Chan SH, . Impairment and disability identity and perceptions of trust, respect, and fairness. JAMA Health Forum. 2023;4(9):e233180. doi:10.1001/jamahealthforum.2023.3180 37738065 PMC10517379

[51] Clark MA, Person SD, Gosline A, Gawande AA, Block SD. Racial and ethnic differences in advance care planning: results of a statewide population-based survey. J Palliat Med. 2018;21(8):1078-1085. doi:10.1089/jpm.2017.0374 29658817

[52] Brown CE, Marshall AR, Snyder CR, . Perspectives about racism and patient-clinician communication among Black adults with serious illness. JAMA Netw Open. 2023;6(7):e2321746. doi:10.1001/jamanetworkopen.2023.21746 37405773 PMC10323709

[53] Baratta J, Zulman D, Verano MR, . Presence for racial justice: disrupting racism through physician-patient communication. Ann Fam Med. 2022;20(20)(suppl 1):2611. doi:10.1370/afm.20.s1.2611 36701757 PMC10549087

