# Supplementary material for: Perceptions of Patient-Clinician Communication Among Adults With and Without Serious Illness
Source: JAMA Netw Open. 2025 Mar 10;8(3):e250365. doi: 10.1001/jamanetworkopen.2025.0365 (PMC11894496; doi:10.1001/jamanetworkopen.2025.0365)
Supplement: Supplement 1. — eTable 1. Outcome Measures and Dichotomization eTable 2. Perceptions of Health Care Communication Experience Outcomes by Serious Illness Status eReferences [file jamanetwopen-e250365-s001.pdf]

Supplementary Online Content

Davila C, Nouri S, Chan SH, et al. Perceptions of patient-clinician communication among adults with and without serious illness. *JAMA Netw Open.* 2025;8(3):e250365. doi:10.1001/jamanetworkopen.2025.0365

eTable 1. Outcome Measures and Dichotomization

eTable 2. Perceptions of Health Care Communication Experience Outcomes by Serious Illness Status

eReferences.

This supplementary material has been provided by the authors to give readers additional information about their work.

© 2025 Davila C et al. *JAMA Network Open*.

eTable 1. Outcome Measures and Dichotomization

**Outcome Measures**	**Survey Question[1]**	**Answer options**	**Dichotomization[a]**
Trust clinicians to do what is right for you	How much of the time do you trust doctors, nurses, and other health professionals to do what is right for you?[b]	Almost none of the time, Some of the time, Most of the time, Almost all of the time	Most of the time, Almost all of the time
Clinicians treat you with dignity and respect	How much of the time do doctors, nurses, and other health professionals treat you with dignity and respect? [b]	Almost none of the time, Some of the time, Most of the time, Almost all of the time	Most of the time, Almost all of the time
Left health care visit feeling unsure about your medicines, what was discussed, or what to do next	How often have you left a health care visit feeling unsure about your medicines, what was discussed, or what to do next?	Never, Rarely, Sometimes, Often, Always	Often, Always
Been afraid to ask questions, speak up, or disagree with clinicians	How often have you been afraid to ask questions, speak up, or disagree with doctors, nurses, and other health professionals because you're worried it may impact your care?	Almost none of the time, Some of the time, Most of the time, Almost all of the time	Most of the time, Almost all of the time
Clinicians talk down to you or make you feel inferior	How often do doctors, nurses, and other health professionals talk down to you or make you feel inferior?[b]	Almost none of the time, Some of the time, Most of the time, Almost all of the time	Most of the time, Almost all of the time
Felt that you were treated unfairly by clinicians in last 12 months	Was there a time in the last 12 months when you felt that you were treated unfairly by doctors, nurses, or other health professionals?[b]	Yes, No, Don't know, Do not wish to answer	Yes

(a) Survey answer choices were transformed from Likert scale format to binary variables, defined as the listed answer choice(s) vs. all other answer choices. These dichotomizations were made to reflect relatively "frequent" vs. "infrequent" occurrences that were felt to be more practical, clinically meaningful, and actionable.
(b) Adapted from Kaiser Family Foundation's "Undefeated Survey on Race and Health."[2]

© 2025 Davila C et al. *JAMA Network Open*.

eTable 2. Perceptions of Health Care Communication Experience Outcomes by Serious Illness Status

Outcome	Response rates, No. (weighted %)[a]			Logistic regression			
	Overall (n = 1847 [100])	Serious illness (n = 363 [18.5])	No serious illness (n = 1484 [81.5]	Unadjusted OR (95% CI)	*P* value	Multivariable AOR (95% CI)[b]	*P* value
Relational goals[c]							
Most of the time or almost always trust clinicians to do what is right	1352 (75.8)	244 (68.7)	1108 (77.4)	0.64 (0.50-0.83)	.001	0.70 (0.53-0.92)	.01
Most of the time or almost always afraid to speak up or ask questions	217 (13.1)	73 (21.4)	144 (11.2)	2.16 (1.58-2.95)	<.001	2.18 (1.55-3.08)	<.001
Have been treated unfairly by clinicians in the past year	287 (15.3)	108 (30.6)	179 (11.8)	3.31 (2.51-4.38)	<.001	3.26 (2.43-4.38)	<.001
Task goals[d]							
Often or always left visit unsure about discussion or next steps	196 (10.7)	69 (18.7)	127 (8.9)	2.35 (1.70-3.26)	<.001	2.30 (1.62-3.27)	<.001
Identity goals[e]							
Most of the time or almost always have been treated with dignity and respect	1563 (85.6)	303 (82.8)	1260 (86.3)	0.77 (0.56-1.06)	.10	0.75 (0.53-1.06)	.10
Most of the time or almost always treated as inferior or talked down to	156 (9.8)	46 (13.2)	110 (9.1)	1.52 (1.05-2.20)	.03	1.90 (1.24-2.91)	.003

[a]Percentages are weighed according to the National Opinion Research Center's statistical weighting methods to account for differences in nonresponse.
[b]Participants with missing predictor or outcome data (<3%) were excluded from multivariable analysis. Fully adjusted models were adjusted for age, gender, race and ethnicity, income, metropolitan area, region, and marital status.
[c]Includes building rapport and establishing mutual trust.
[d]Includes disclosing medical information, such as prognosis.
[e]Includes tailoring conversations to individual needs of patient and family.

© 2025 Davila C et al. *JAMA Network Open*.

eReferences.

1. Davila C, Chan SH, Gosline A, et al. Online Forums as a Tool for Broader Inclusion of Voices on Health Care Communication Experiences and Serious Illness Care: Mixed Methods Study. *Journal of Medical Internet Research*. 2023;25(3):e48550-e48550. doi:10.2196/48550
2. Hamel L, Lopes L, Munano C, Artiga S, Brodie M. KFF/The Undefeated Survey on Race and Health. Kaiser Family Foundation. https://www.kff.org/racial-equity-and-health-policy/report/kff-the-undefeated-survey-on-race-and-health/

© 2025 Davila C et al. *JAMA Network Open*.
