# Supplementary material for: Perceptions of Patient-Clinician Communication Among Adults With and Without Serious Illness
Source: JAMA Netw Open. 2025 Mar 10;8(3):e250365. doi: 10.1001/jamanetworkopen.2025.0365 (PMC11894496; doi:10.1001/jamanetworkopen.2025.0365)
Supplement: Supplement 2. — Data Sharing Statement [file jamanetwopen-e250365-s002.pdf]

Data Sharing Statement

Davila. Perceptions of Patient-Clinician Communication Among Adults With and Without Serious Illness. *JAMA Netw Open*. Published March 10, 2025.
doi:10.1001/jamanetworkopen.2025.0365

Data
Data available: No

Additional Information
Explanation for why data not available: This patient-related information is not available for sharing. Upon request, a codebook can be made available to replicate analyses.
